# Accuracy and precision of the spinal instability neoplastic score (SINS) for predicting vertebral compression fractures after radiotherapy in spinal metastases: a meta-analysis

**DOI:** 10.1038/s41598-021-84975-3

**Published:** 2021-03-10

**Authors:** Young Rak Kim, Chang-Hyun Lee, Seung Heon Yang, Seung-Jae Hyun, Chi Heon Kim, Sung Bae Park, Ki-Jeong Kim, Chun Kee Chung

**Affiliations:** 1 Department of Neurosurgery, Seoul National University Hospital, Seoul, Republic of Korea; 2 Department of Neurosurgery, Spine Center, Seoul National University Bundang Hospital, 82, Gumi-Ro 173, Bundang-Gu, Seongnam, Gyeonggi 13620 Republic of Korea; 3 Department of Neurosurgery, Seoul National University College of Medicine, Seoul, Republic of Korea; 4 Department of Neurosurgery, Seoul National University Boramae Hospital, Seoul, Republic of Korea; 5 Department of Brain and Cognitive Sciences, Seoul National University College of Medicine, Seoul, Republic of Korea

**Keywords:** Spine regulation and structure, Bone cancer, Cancer epidemiology, Cancer, Neurology

## Abstract

Radiotherapy has played an important role in the treatment of spinal metastases. One of the major complications of radiotherapy is vertebral compression fracture (VCF). Although the spinal instability neoplastic score (SINS) was developed for evaluating spinal instability in patients with spinal metastases, it is also commonly used to predict VCF after radiotherapy in patients with spinal metastases. However, its accuracy for predicting radiotherapy-induced VCF and precision remain controversial. The aim of this study was to clarify the diagnostic value of the SINS to predict radiotherapy-induced VCF and to make recommendations for improving its diagnostic power. We searched core databases and identified 246 studies. Fourteen studies were analyzed, including 7 studies (with 1269 segments) for accuracy and 7 studies (with 280 patients) for precision. For accuracy, the area under the summary receiver operating characteristic curve was 0.776. When a SINS cut-off value of 7 was used, as was done in the included studies, the pooled sensitivity was 0.790 and the pooled specificity was 0.546. For precision, the summary estimate of interobserver agreement was the highest dividing 2 categories based on a cut-off value of 7, and the value was 0.788. The *body collapse* showed moderate relationship and precision with the VCF. The lytic tumor of *bone lesion* showed high accuracy and fair reliability, while *location* had excellent reliability, but low accuracy. The SINS system can be used to predict the occurrence of VCF after radiotherapy in spinal metastases with moderate accuracy and substantial reliability. Increasing the cut-off value and revising the domains may improve the diagnostic performance to predict the VCF of the SINS.

## Introduction

Spinal metastases can result in neoplastic spinal instability, which is defined as movement-related pain, deformities, or neurological compromise under physiological loads, and spinal instability frequently has detrimental effects on patients’ quality of life by inducing vertebral body fractures^1^. Conventional radiotherapy (CRT) has traditionally played an important role in pain palliation, and spine stereotactic body radiotherapy (SBRT) has demonstrated safety and efficacy for the treatment of spinal metastases, with encouraging results both as a primary modality and in the setting of repeated irradiation after conventional radiation treatment failure^2^. The major complications of radiotherapy are radiation myelopathy and vertebral compression fracture (VCF)^3^. Previously published papers reported that the radiotherapy-induced VCF occurred in 11–41% of cases^2,4–6^. The only consistent predictor of the VCF in multivariable analyses was found to be lytic tumors^3–6^.

The Spine Oncology Study Group developed the spinal instability neoplastic score (SINS) in 2010 to assess the degree of spinal (in)stability caused by metastatic diseases, as presented in Table 1^7–9^. The score consists of the sum of 5 radiographic parameters and 1 clinical parameter, which results in a summed score between 0 and 18 points^9^. The total score is then divided into 2 categories (stable, 0–6 points and unstable, 7–18 points) or 3 categories of spinal stability (stable, 0–6 points; impending/potentially unstable, 7–12 points; and unstable, 13–18 points)^9^. Because the SINS provides a common language to discuss spinal instability, its use can improve the uniform reporting of spinal instability in the published literature and communication among oncologists, radiologists, and spine surgeons^8,10–12^. In recent years, the SINS has emerged as the most widely accepted instrument for classifying the stability of metastatic vertebral segments^13^.

**Table 1:** Spinal instability neoplastic score (SINS).

SINS component	Score
**Location**	
Junctional (occiput-C2, C7–T2, T11–L1, L5-S1)	3
Mobile segment (C3–C6, L2–L4)	2
Semirigid (T3–T10)	1
Rigid (S2–S5)	0
**Pain**	
Yes	3
Occasional pain but not mechanical	1
Pain-free lesion	0
**Bone lesion**	
Lytic	2
Mixed (lytic/blastic)	1
Blastic	0
**Spinal alignment**	
Subluxation/translation	4
De novo deformity (kyphosis/scoliosis)	2
Normal alignment	0
**Body collapse**	
> 50% collapse	3
< 50% collapse	2
No collapse with > 50% body involved	1
None of above	0
**Posterolateral involvement of spinal elements**	
Bilateral	3
Unilateral	1
None	0

Binary scale (0–6, stable; 7–18, unstable).

Tertiary scale (0–6, stable; 7–12, potentially unstable; and 13–18, unstable).

Although the SINS was developed for evaluating spinal instability in patients with spinal metastases, it is also used in other scenarios, such as VCF after radiotherapy and instability associated with a primary bone tumor^14,15^. Several previous studies have reported that the SINS may be a useful tool for predicting VCF in patients who have undergone radiotherapy, and that it had substantial to excellent interobserver and intraobserver reliability^16–19^. Other studies have reported that the SINS score was not predictive of new VCFs after radiotherapy^3,6,20^. Another study reported that the statistical power of the accuracy of SINS to predict radiotherapy-induced VCF was significant only in the univariate analysis, but not in the multivariate analysis^21^. Regarding precision, some researchers questioned the results of those studies, as some of the studies were authored by a co-developer of the SINS^11,18,22^. Therefore, the accuracy and precision of the SINS require objective evaluation by independent researchers.

The primary purpose of this study was to evaluate the accuracy of the SINS for predicting radiotherapy-induced VCF and to evaluate the scores assigned for the 6 domains of the SINS by performing a meta-analysis of diagnostic test accuracy. The secondary aim was to evaluate the precision of the SINS overall and for each domain through a meta-analysis of summary estimates.

## Materials and methods

### Search strategy and study selection criteria

We performed a comprehensive literature search to identify studies that applied the SINS in cases of spinal metastases, according to the Preferred Reporting Items for Systematic Reviews and Meta-Analyses (PRISMA) guidelines. The searched databases included PubMed, Embase, Web of Science, and the Cochrane Database from inception to January 2020. The search terms used were “spinal instability neoplastic score” AND “spine” (or “spinal”) AND “metastasis” (or “metastases”). We also examined the references of all included papers to find other relevant articles. There were no language restrictions on study eligibility, and only the largest study was included in the case of overlapping study populations. We excluded duplicated studies, narrative reviews, letters, editorials, comments, and case reports. Studies were also excluded if they included primary tumors (e.g., lymphoma), used the SINS to predict other outcomes (e.g., survival); or did not report target outcomes. The PRISMA checklist has been submitted to the journal as an attachment to this article (see Supplementary Table S1).

### Study eligibility criteria

Two independent reviewers (Y.R.K. and C.H.L.) assessed the eligibility of all the studies retrieved from the databases and performed quality assessments. Any disagreement between the reviewers was resolved through a discussion. We used 2 methods of meta-analysis. First, a meta-analysis of diagnostic test accuracy was performed to evaluate the accuracy of radiotherapy-induced VCF prediction. We assessed the quality of the studies using the outlined component approach for diagnostic accuracy studies with the Quality Assessment of Diagnostic Accuracy Studies 2 (QUADAS-2) tool^23^. We systematically reviewed published studies on the basis of the following criteria: (1) studies that used the SINS to predict VCFs in patients with spinal metastases; (2) studies that reported the numbers of patients for 2 or 3 SINS categories and the number of VCFs; and (3) studies that used data with sufficient information to assess true-positive (TP; fracture in the unstable group), true-negative (TN; no fracture in the stable group), false-positive (FP; fracture in the stable group), and false-negative (FN; no fracture in the unstable group) cases.

Second, a meta-analysis of summary estimates was performed to evaluate the interobserver reliability of the overall score, categories, and each domain of SINS (pain, location, bone lesion, alignment, collapse, and posterolateral involvement). For evaluating interobserver reliability, we assessed the bias risk using a modified form of the Newcastle–Ottawa Scale for non-randomized studies^24,25^. Studies were included if they contained a point estimate of the Cohen or Fleiss kappa (*κ*) value and 95% confidence intervals (CIs).

### Data synthesis and analysis

#### Accuracy for predicting radiotherapy-induced VCF

The retrieved data included the following items: name of the first author; year published; patient demographics; numbers of TP, TN, FP, and FN cases; and the numbers of patients with and without fractures and their scores for the 6 individual SINS domains. Test accuracy was calculated using a summary receiver operating characteristic (SROC) model, the area under the curve (AUC), and the index Q value. With respect to the AUC, a value of 0.5 was considered non-informative; a value of > 0.5 but ≤ 0.7 was considered less accurate; a value of > 0.7 but ≤ 0.9 was considered moderate; a value of > 0.9 but < 1 was considered very accurate; and a value of 1 was considered perfect^26,27^. To perform a meta-analysis of diagnostic test accuracy using all the available studies that reported more than one threshold value, we created 2-by-2 tables for each value from the included studies. Statistical analyses were performed using R version 3.6.3 (R Foundation for Statistical Computing, Vienna, Austria). For the subgroup analysis, each domain (categorical variables) of SINS was analyzed using Spearman rank-order correlation analysis to evaluate the relationship between the incidence of VCF and each domain of SINS. As a post-hoc test, we compared two adjacent classes (ordinal variables) of the 6 domains of SINS using odds ratios and the chi-square test. Review Manager version 5.3 Cochrane Collaboration, Oxford, UK) was used for the QUADAS-2 tool, coupled forest plot, and SROC plots.

### Precision of the SINS

The collected data included the *κ*-values and 95% CIs of the overall SINS; the SINS used as a categorical measure with 2 categories (< 7 vs. ≥ 7) or 3 categories (0–6 vs. 7–12 vs. 13–18); and the 6 individual domains of SINS. The summary estimate was calculated as the weighted mean of the reported number of participants and pooled variance. Pooled estimates were categorized as follows according to the method of Landis and Koch: near perfect (0.81–1.00), substantial (0.61–0.80), moderate (0.41–0.60), fair (0.21–0.40), and slight (0.00–0.20)^28^. Heterogeneity was considered significant when the *I*^2^ value was > 50%^29^. A random-effects model was used depending on the study design and heterogeneity of the studies included. We assessed publication bias by visually inspecting the funnel plots and calculating the p-value (one-sided) for Egger’s intercept^30^. Data were analyzed using the Comprehensive Meta-Analysis software version 3.3 (Biostat, Inc., Englewood, NJ, USA).

## Results

### Search results for relevant studies

The initial search identified 246 articles, from which 143 duplicated articles were excluded. Among the 103 remaining articles, 23 were excluded because they were case reports, review articles, letters, or technical notes. Some papers dealt with other tumors (n = 17) such as myeloma, granuloma, or lymphoma, and 12 were research on metastatic epidural spinal cord compression. The remaining 51 studies were subjected to full-text review and another 37 were excluded. The reasons for exclusion of these studies were no data on accuracy or precision (n = 28) or no prediction of VCF (n = 9). Finally, we identified a total of 14 observational studies. The detailed results of the selection process are shown in Supplementary Fig. S1.

Seven of the included studies^3,6,21,31–34^ evaluated accuracy and included 798 patients and 1269 spinal segments, and the other 7 studies^11,16–18,22,35,36^ evaluated precision and included 280 patients (Table 2). Four studies^3,6,21,31^ dealt with patients who underwent SBRT, the other studies^32–34^ evaluated patients who underwent CRT. The primary regions of cancer were the kidney, breast, lung, and colorectum for the studies evaluating accuracy, and the kidney, breast, lung, and prostate for the studies analyzing precision.

**Table 2:** Baseline characteristics of enrolled studies.

	Author	Study region	Primary cancer (major organs)	Number of patients	Mean age (SD)	Mean follow-up (SD)	Evaluator (number)
Accuracy	Cunha 2012^6^	Canada	Multiple (Kidney, Breast, Lung)	90	57.0 (18.4)	7.4 (9.4)	
	Sahgal 2013^3^	US	Multiple (Kidney, Breast, Lung)	252	57.6 (18.4)	11.5 (28.8)	
	Thibault 2014^31^	Canada	Renal cell cancer	37	63.0 (12.5)	12.3 (13.8)	
	Thibault 2015^21^	US, Canada	Renal cell cancer	116	60.2 (13.9)	8.0 (19.4)	
	Aiba H 2016^34^	Japan	Non-small cell lung cancer	47	67	10.2 (13.7)	
	Shi 2018^32^	US	Multiple (Breast, Lung)	203	60.0 (18.9)	5.9	
	Lee 2018^33^	Korea	Colorectal Cancer	53	61.0 (10.7)	10 (24.5)	
Precision	Fourney 2011^11^	World	N/D	30	N/D		SOSG (24)
	Teixeira 2013^17^	Brazil	N/D	40	N/D		Physician (17) Spine surgeons (10)
	Campos 2014^16^	N/D	Multiple (Kidney, Breast, Lung)	30	65.0 (14.3)		Spine surgeon (3) Physician (3)
	Fisher 2014a^22^	World	Multiple (Lung, Prostate, Breast)	30	N/D		Radiologist (37) Spine surgeon (11)
	Fisher 2014b^18^	World	Multiple (Lung, Prostate, Breast)	30	N/D		Radiation oncologist (33) Spine surgeon (11)
	Arana 2016^36^	Spain	Multiple (Breast, Prostate, Lung)	90	N/D		Spine surgeon (30)
	Fox 2017^35^	Canada	N/D	30	N/D		Resident (23) Fellow (2)

*SD* standard deviation, *N/D* not described, *SOSG* the spine oncology study group.

### Accuracy of the SINS in predicting VCFs

**Figure 1:**
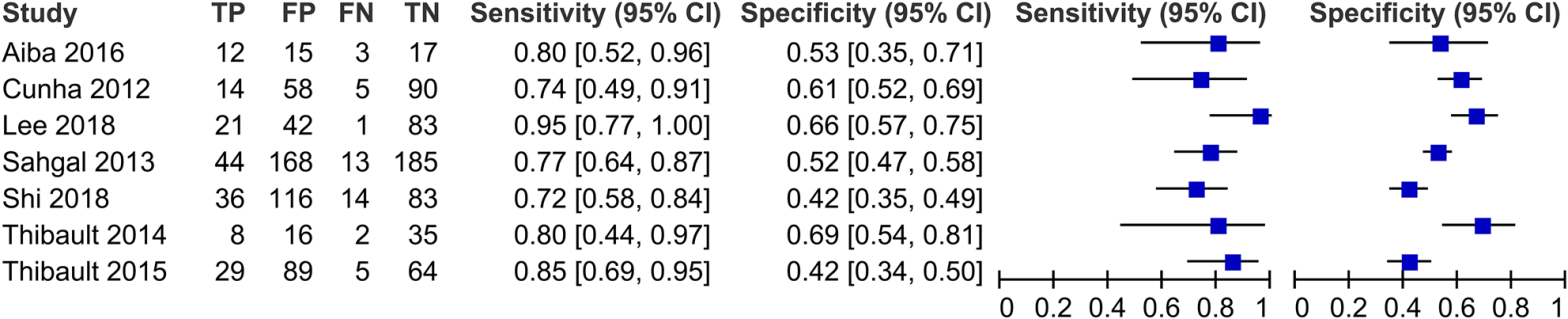
Coupled forest plots of diagnostic accuracy. Sensitivity and specificity of the spinal instability neoplastic score (SINS) for predicting the occurrence of vertebral compression fractures after radiotherapy in spinal metastases. This figure was drown using the Review Manager version 5.3.

**Figure 2:**
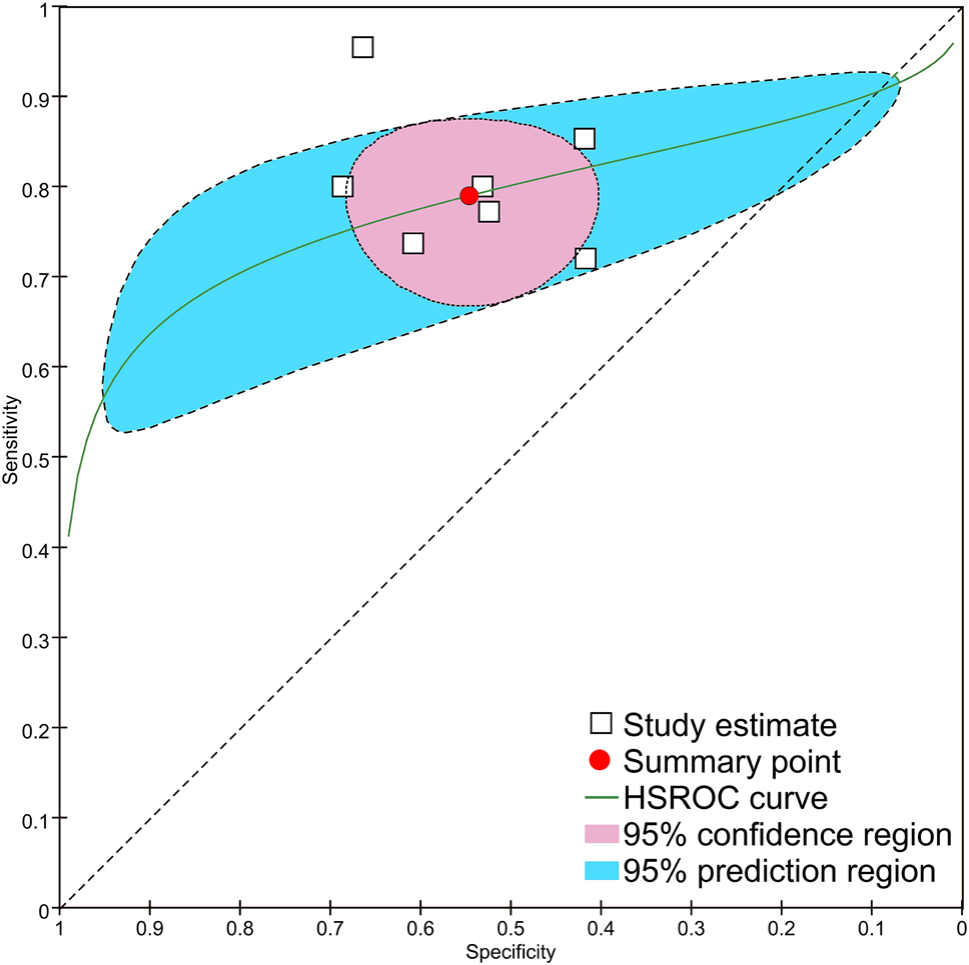
Summary of the receiver operating characteristic (SROC) plot of the spinal instability neoplastic score (SINS). The pooled sensitivity, specificity, and SROC curve for the prediction of vertebral compression fracture using the SINS are depicted. The area under the SROC curve (AUC) was 0.776. If the cut-off value was 7, the pooled sensitivity was 0.790 (95% confidence interval [CI] 0.723–0.843) and the specificity was 0.546 (95% CI 0.462–0.627) as indicated by the red point. The 95% confidence region is shown in pink. This figure was drown using Review Manager version 5.3.

Using data from the included studies, a 2 × 2 table was created based on VCF using a SINS cut-off of 7. We used the same classification criteria as the included studies and defined TP as a SINS of ≥ 7 with a VCF, FP as a SINS of ≥ 7 without a VCF, FN as a SINS of < 7 with a VCF, and TN as a SINS of < 7 without a VCF. Coupled forest plots showed the sensitivities and specificities of each study (Fig. 1). An SROC plot was created using data from 7 studies (Fig. 2). The AUC of the SROC plot was 0.776, which suggests that the SINS is a moderately reliable test for predicting VCF. When the cut-off value was 7, the summary estimate of the SINS predicted VCF after radiotherapy in patients with spinal metastases with a pooled sensitivity of 0.790 (95% CI 0.723–0.843) and a pooled specificity of 0.546 (0.462–0.627).

**Figure 3:**
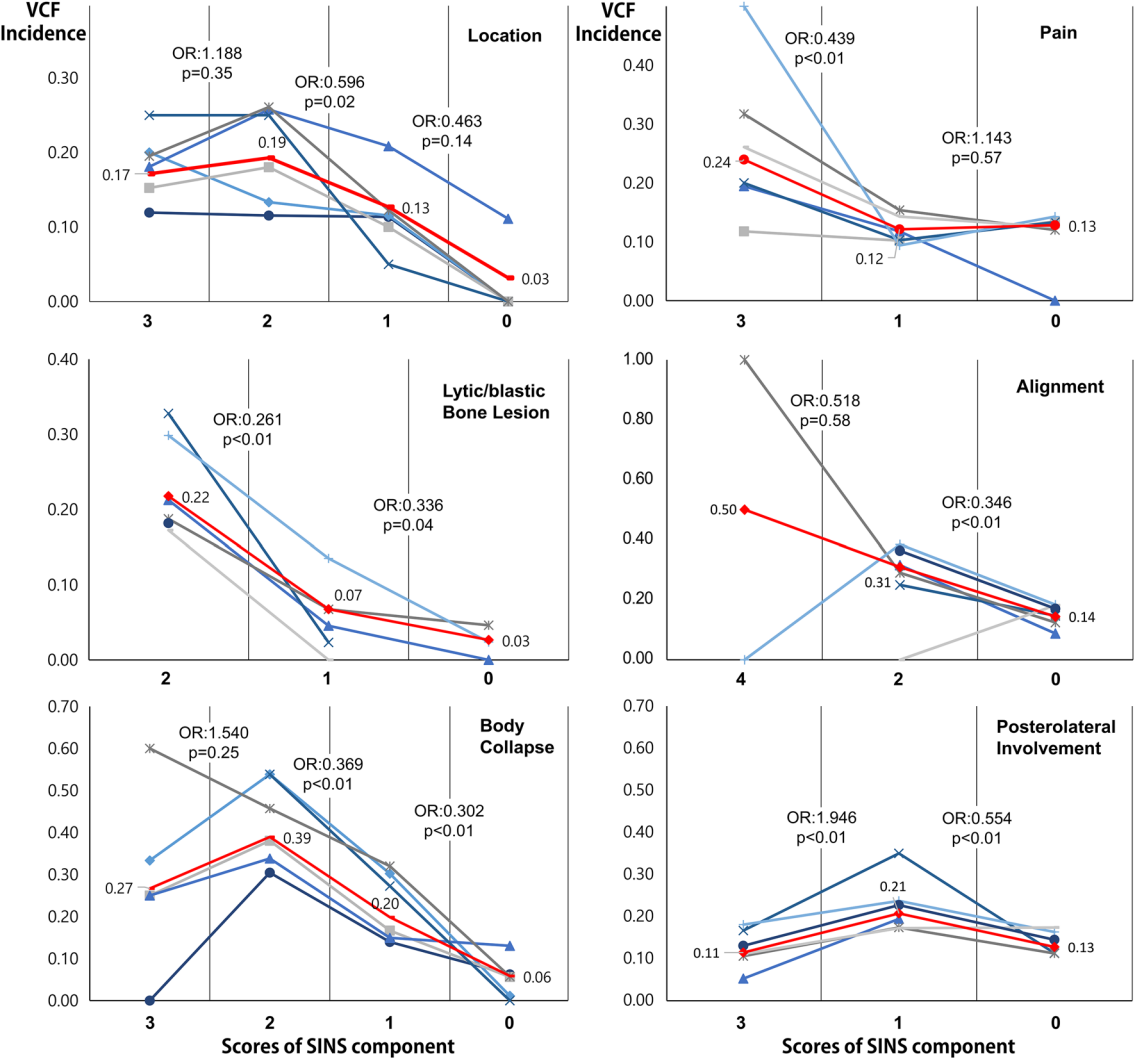
Incidence of vertebral compression fracture (VCF) after radiotherapy according to the score of each domain of the spinal instability neoplastic score (SINS). The red line shows the mean incidence rate. The lines with other colors represent each of the included studies. The lytic lesion (score 2) of bone lesion showed more frequent than the other lesions. In terms of the body collapse domain, the incidence decreased significantly as the score decreased, displaying high accuracy. Some domains and levels showed an increasing incidence of VCF despite decreasing scores. This figure was drown using Microsoft Excel version 2016.

A subgroup analysis was performed to assess the correlation between the scores for the 6 SINS domains and the incidence of VCF (Fig. 3, Table 3). The domain of body collapse showed a moderate relationship (correlation coefficient, 0.333; p < 0.001) and was closely correlated with VCF for scores of 0–2. However, the incidence of VCF with a > 50% collapse (score 3) was lower than that of VCF with a < 50% collapse (score 2), even as the score increased. In the bone lesion domain, the incidence of VCF decreased as the score decreased, with a weak correlation (correlation coefficient, 0.218; p < 0.001) between VCF and the bone lesion score. A low bone lesion score in the SINS indicates that a lytic tumor (score 2) is associated with a high risk of VCF after radiotherapy. There was a slight difference between mixed (score 1) and blastic (score 0) tumors. The other domains (location, pain, alignment, and posterolateral involvement) showed negligible relationships with VCF in the correlation analysis. Spinal alignment showed a difference between kyphosis/scoliosis (score 2) and normal (score 0). There were only 2 cases of score 4 (subluxation/translation) of spinal alignment, and an analysis showing statistical significance was not possible for a comparison between scores 2 and 4. A correlation between the location of metastases and incidence of VCF was only found for mobile (score 2) and semi-rigid (score 1) metastases. Patients with occasional pain (score 1) showed fewer VCFs than those with persistent pain (score 3) and a similar incidence of VCF as the pain-free group (score 0). Unilateral posterolateral involvement (score 1) was associated with a higher incidence of VCF than bilateral involvement (score 3) or no involvement (score 0).

**Table 3:** Spearman rank-order correlation analysis between VCF and each domain of SINS.

Domain	Number of patients	Correlation coefficient with VCF	Relation strength of the variables^a^	P
Location	1,222	0.070	No or negligible relationship	0.015
Pain	1,222	0.121	No or negligible relationship	< 0.001
Bone lesion	1,222	0.218	Weak relationship	< 0.001
Alignment	1,222	0.127	No or negligible relationship	< 0.001
Body collapse	1,222	0.333	Moderate relationship	< 0.001
Posterolateral involvement	1,222	0.007	No or negligible relationship	0.813

^a^Interpretation of Spearman rank-order correlation coefficient was adapted from Dancey and Reidy^37^.

### Precision of the SINS

**Figure 4:**
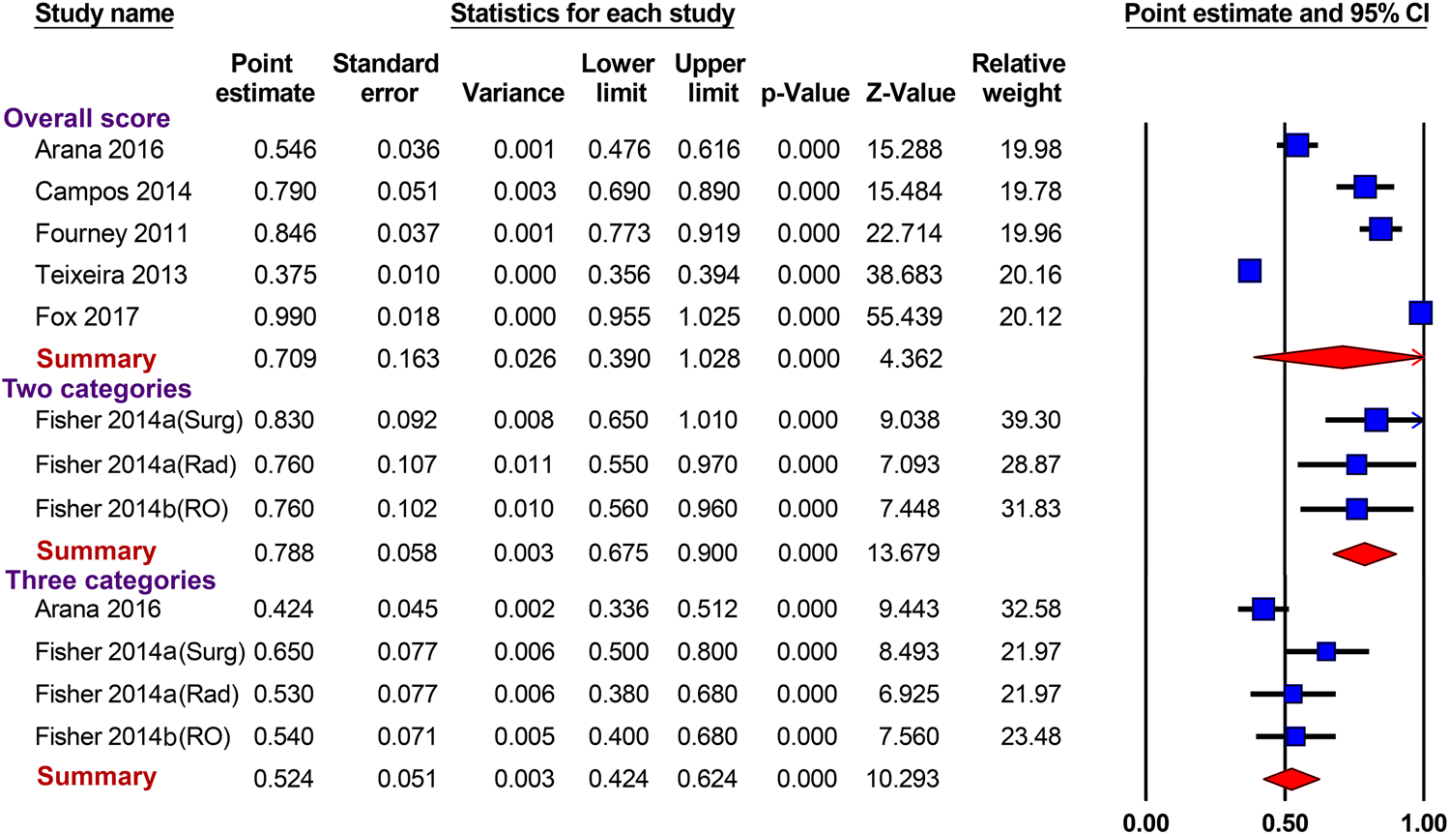
Forest plots of interobserver reliability (*κ*-values). The summary estimates of the interobserver reliability were 0.709 for the overall score, 0.788 when 2 categories were used, and 0.524 when 3 categories were used. Despite the small number of studies, the results of the studies were highly consistent, with a low standard deviation. This figure was drown using the comprehensive Meta-Analysis software version 3.3.

The interobserver agreement (*κ*-value) of the SINS was calculated in 7 studies. The summary estimates for the overall score of the SINS, 2 categories, and 3 categories was as follows: 0.709 (95% CI 0.390–1.028), 0.788 (95% CI 0.675–0.900), and 0.524 (95% CI 0.424–0.624), respectively (Fig. 4). The use of SINS with 2 categories (stable vs. unstable) showed substantial agreement and the highest interobserver reliability. A subgroup analysis of interobserver reliability for each domain was performed, and the results are presented in Table 4. Location showed near-perfect precision (*κ* = 0.833 [95% CI 0.825–0.841]). Alignment, body collapse, and posterolateral involvement demonstrated moderate agreement, with *κ*-values of 0.480 (95% CI 0.405–0.556), 0.479 (95% CI 0.421–0.537), and 0.450 (95% CI 0.408–0.493), respectively. For the pain domain, reliability was substantial (*κ* = 0.680 [95% CI 0.416–0.945]). The reliability of the bone lesion domain was fair (*κ* = 0.280 [95% CI 0.209–0.350]).

**Table 4:** Precision of 6 domains of the Spine Instability Neoplastic Score (SINS).

Domain	Studies	Number of patients	Summary of interobserver reliability (κ-value, interpretation)	95% Confidence interval	*I*^*2*^	Egger test (p, 1-tailed)
Location	^11,17,35,36^	190	0.833 (near perfect)	0.825–0.841	99%	0.39
Pain	^11,17,35^	100	0.680 (substantial)	0.416–0.945	99%	0.42
Bone lesion	^11,17,35^	100	0.280 (fair)	0.209–0.350	96%	0.18
Alignment	^11,17,35^	100	0.480 (moderate)	0.405–0.556	96%	0.45
Body collapse	^11,17,35^	100	0.479 (moderate)	0.421–0.537	94%	0.19
Posterolateral involvement	^11,17,35^	100	0.450 (moderate)	0.408–0.493	95%	0.07

### Publication bias

The results of the QUADAS-2 analysis are summarized in terms of risk of bias and concerns regarding applicability in Supplementary Fig. S2^23^. Most of the included studies predicted VCF after radiotherapy in patients with spinal metastases. One study predicted skeletal-related events, which included pathologic fractures, the need for surgery, bone irradiation, spinal compression, and hypercalcemia^34^. For evaluating interobserver reliability, the results of the quality assessment were acceptable. One study revealed that evaluations using the SINS were performed by residents and fellows, while others reported the evaluations were performed by a specialist^35^.

## Discussion

When predicting the occurrence of VCF after radiotherapy based on a SINS score of 7 points in patients with spinal metastases, the accuracy of the tool was moderately significant (AUC, 0.776), and the interobserver reliability showed substantial agreement (*κ* = 0.799). Among the 6 SINS domains, body collapse domain was moderately correlated with occurrence of VCF and bone lesion was weakly correlated; however, the other domains showed insignificant relationships with the incidence of VCF. In aspect of precision, bone lesion domain showed fair interobserver reliability (*κ* = 0.28), while location displayed near-perfect reliability (*κ* = 0.83). Although the overall accuracy and precision were acceptable, some domains showed high accuracy and low precision, or high precision and low accuracy.

This meta-analysis revealed that the diagnostic power of the SINS in predicting radiotherapy-induced VCF was moderate. All of the included studies used a SINS score of 7 to distinguish between low and high risk for VCF, and we used the same cut-off value and integrated summary estimates. When the cut-off value was 7, the pooled sensitivity and specificity were 0.79 and 0.54, respectively, which demonstrated substantially low specificity. SROC analyses provide important information about diagnostic test performance; the closer the apex of the curve is to the upper left corner, the greater the discriminatory ability of the test^38^. Considering the shallow slope of the curve, the best cut-off value (the closest point to the upper top corner) was mildly greater than 7 in the SROC curve. At an ideal threshold, the sensitivity and specificity would be approximately 0.7 and 0.8, respectively. The summary estimate of the interobserver reliability of the 2 SINS categories showed substantial agreement. Although only 2 studies (written by the same corresponding author) evaluated the precision of the SINS using 2 categories^18,22^, similar results for precision were observed for the overall SINS score in studies published by 5 different authors.

### Subgroup analysis of 6 SINS domains

When 6 domains of SINS were analyzed, the body collapse domain also showed significance. The incidence of VCF at > *50% collapse* (score 3) was 27%, which was substantially lower than that *at* < *50% collapse* (score 2; 39%). The reason for this may be that the lesions that already showed extensive collapse were unlikely to develop additional fractures. The incidence of VCF at > *50% collapse* was similar to the incidence in cases of *no collapse with* > *50% involvement* (score 1; 20%). If the groups with scores of 3 and 1 in the body collapse domain were combined and the number of levels decreased from 4 (3, 2, 1, and 0) to 3 (2, 1, and 0), the accuracy and reliability would increase. This finding may mean that highly (> 50%) collapsed lesions can be more stable than slightly collapsed lesions. The reason for this may be that highly compressed lesions have no more space to collapse due to the compressed bone marrow.

The score for the bone lesion domain revealed a weak relationship with the incidence of radiotherapy-induced VCF. The incidence of VCF was 22% in the lytic group (score 2), 7% in the mixed group (score 1), and 3% in the blastic group (score 0). The mixed lesion group showed a similar VCF incidence rate to that of the blastic group, so it may be better to combine these 2 groups. Interobserver reliability of bone lesion demonstrated fair (*κ* = 0.28), and the reason for its fair reliability may be that clinicians may have difficulty distinguishing between mixed (lytic/blastic) and lytic and blastic lesions.

Alignment was an accurate predictor in a comparison between de novo deformity (kyphosis/scoliosis) (score 2) and normal alignment (score 0), whereas the VCF incidence was not significantly different between subluxation/translation (score 4) and kyphosis/scoliosis (score 2). This was likely due to the presence of only 2 of 1222 patients with a score of 4. Therefore, eliminating the subluxation/translation level because of the scarcity of cases may improve the accuracy and reliability of the SINS test.

The incidence of VCF in cases with unilateral posterolateral involvement (score 1) was 21%, significantly higher than the 11% in the bilateral involvement group (score 2) and 13% in the cases without any involvement (score 0). Although all 5 studies showed the same trend, none proposed an explanation for this trend. A further investigation into the relationship between VCF and posterolateral involvement is needed to clarify this result.

The location domain showed near-perfect (*κ* = 0.83) agreement in terms of interobserver reliability, and using this domain may be one way of increasing the overall reliability of the SINS. However, the incidence rates of VCF at the different levels were 17%, 19%, and 13% in junctional, mobile, and semirigid sections, respectively, demonstrating that different locations were not associated with a significantly different risk of VCF.

Pain, the only clinical SINS domain, showed a substantial agreement in interobserver reliability; however, the reliability might have been overestimated because the evaluators did not examine the patients themselves, but rather reviewed their medical records and evaluated the scores. The VCF incidence rates were 24%, 12%, and 13% in patients with persistent pain (score 3), occasional pain (score 1), and pain-free lesions (score 0), and no statistically significant difference was found in VCF incidence between the scores of 1 and 0. Although the VCF incidence showed a substantial difference between scores 3 and 1, the incidence in the group with a score of 3 was quite variable, ranging from 12 to 50% among the studies. Owing to the difficulty of fully distinguishing the pain caused by spinal metastases from other kinds of pain, disability indexes may be more accurate parameters.

### Limitations

This study has several limitations that need to be addressed. First, the SINS was developed not to predict radiotherapy-induced VCF, but to evaluate spinal instability. Therefore, the accuracy of the SINS presented in this study is limited to the prediction of VCF in the specific scenario of radiotherapy-induced VCF, and the findings of this study have no implications for the accuracy of the SINS when used for its original purpose. Second, there was substantial heterogeneity in radiotherapy, patients, and evaluators. The pooled data were very heterogeneous, since different radiation protocols were reported in each study (CRT or SBRT). The radiation doses and fractionation schemes are very important, and may affect the VCF. For the analysis of accuracy, osteolytic metastases were included. Precision may be affected by the professional expertise and level of experience of the evaluators^39^. The evaluations using the SINS were performed in this meta-analysis by specialists in the fields of spine surgery, radiology, and radiation oncology, as well as trainees such as residents and fellows, which may have reduced the precision. Nonetheless, the SINS was developed to facilitate communication between non-surgeons and surgeons, and evaluations conducted by various health-care professionals are therefore representative of the real-world use of this instrument. Third, the SINS was introduced as a tool for clinicians to recommend surgery before the onset of severe disability caused by VCF. However, this scoring system can miss patients with VCF due to a low disability level or lack of obvious VCF because of a high disability level, such as being in a bedridden state. The clinical implications of this tool should be investigated to better understand and use the SINS with caution, as the disability index at baseline was not considered. Fourth, 6 of the 7 included studies for accuracy enrolled patients who underwent SBRT or CRT^3,6,21,31–33^. The reported median dose was 24 Gy for SBRT and 30 Gy for CRT. A high radiation dose may affect the incidence of VCF. Although this was an uncontrolled confounding factor that may have created bias, the target radiation dose was usually constant in the included studies, and follow-up without local treatment for spinal lesions after the diagnosis was almost impossible. Finally, pain as a SINS domain might not be consistently evaluated. Evaluators need to distinguish mechanical pain that improves with recumbency or pain with movement or spinal loading from pain due to degenerative disease or trauma. We conducted this meta-analysis under the assumption that the studies distinguished these types of pain correctly. If the pain data of the included studies are uncertain, the results of a meta-analysis using these data need to be interpreted with caution.

## Conclusion

The system involving a binary categorization based on the SINS can be used to predict the occurrence of VCF after radiotherapy in spinal metastases with moderate accuracy and substantial reliability. The body collapse showed moderate accuracy and precision with the VCF. The lytic bone lesion was a risk factor of VCF, but bone lesion domain had only fair reliability, whereas location was nearly perfectly reliable, but was inaccurate. Raising the cut-off value to above 7 and revision of some domains may help improve diagnostic accuracy.

## Supplementary Information

Supplementary Information.
